# Home Department

**Published:** 1886-09

**Authors:** 


### HOME DEPARTMENT.

**Bran** or oatmeal water should be used when bathing children suffering with scarlet fever.

**Tomato Salad.**—Peel and slice six large tomatoes; take one teaspoonful of oil, one of vinegar, a teaspoonful each of mustard, salt and pepper; mix and pour over the tomatoes.

**Puff Pudding.**—One pint of boiling milk and 9 tablespoonfuls of flour, mixed first with a little cold milk. When cold add a little salt and four well-beaten eggs and bake in a buttered dish. Serve as soon as it is done.

**Discolored marble** can often be restored by rubbing with pumice stone. For an effective polish take one half ounce of magnesia, two ounces oxalic acid, one pint of warm rain water. Polish with woolen cloths. Be careful of the acid, however.

**Fruit stains** may easily be removed from white or light goods by placing the article in a bath of clear, boiling water before it has been wet with soap suds; let the goods remain till quite cool, wring, and if still the stains show immerse again in hot water.

**Iron Rust in Dry Goods.**—Iron rust is readily removed by equal parts of common salt and cream of tartar moistened with water and applied to the stained spots and placed in the sunshine. Moisten as it becomes dry, for two or three hours.

**German Cakes.**—One egg, 7 ounces of butter, 4 ounces of powdered sugar, 10½ ounces of flour, 1 tablespoonful of molasses. Mix without adding the wetting, and roll out; sprinkle cinnamon and sugar on top, roll again thinner, and cut out into little cakes.

**Cheap Cake.**—Two cups of molasses, 1½ cups of boiling water, 2 tablespoonfuls (not heaped) of tried-out beef suet, or a piece of butter the size of an egg, 3 heaped teaspoonfuls of baking powder, spices to taste, and a cup of raisins and currants. Add flour enough to make a soft batter, and bake in loaves.

**Batter For Fritters.**—Beat up 1 tablespoonful of brandy and a little cold water, with the yolk of 1 egg; add a pinch of salt, then work in sufficient flour to make, with the addition of more water, as much batter as will be needed. It should be of the consistency of thick cream. Just before using beat the whites of 2 eggs to a stiff froth and mix them lightly but effectually with the butter.

**To Put up Jelly.**—Jellies are usually put in tumblers, sometimes with tin covers, sometimes without. Cut soft brown paper into rounds half an inch larger than the tumbler. Dip them into water and flour mixed to the consistency of thick milk. Drain and spread them on the top. Rub them down smoothly and they will be air tight. Label with the name and date of the jelly.

**French Apple Fritters.**—Peel 3 large apples, core them with an apple corer, and cut them across in slices rather less than ½ inch thick; put them in a flat dish with ½ tumbler of strong cider and strew them plentifully with powered loaf sugar; let them remain covered for a couple of hours, then take each piece separately, dip it in batter so that it is well covered with it, and fry a golden color in hot lard. Lay the fritters in front of the fire, and when all are done pile them up on a napkin, shake plenty of powdered sugar over and serve.

**Preserved Citron Melon.**—Take three medium sized melons, six lemons, one pound preserved green ginger root; peel, slice about a half inch thick and seed the melons. Weigh, and take the same weight in fine white sugar. Boil the slices in water until they are tender; take them out on a flat dish to cool. Then put the sugar in the same quantity of water for the syrup. Slice the lemons thin in cold water and let them cook tender. Then turn the water on the syrup and boil until clarified, with the ginger sliced thin. Put in the melon and boil until scalded through. Then take it out on a dish to cool, and after it is cool pour the syrup over it. It doesn't need to be put in preserving jars. Put it in an earthenware jar, cover it over, and it will keep all right in a cool place without moulding.

**Clam Chowder.**—Take the kettle in which your chowder is to be made and place within a half pound of pork which you have cut into inch pieces. Let this fry until the fat is all drawn out, but be very careful not to let it burn. Remove every piece of pork and place in the fat remaining, two onions which you have peeled and chopped fine. When they are all well cooked, remove the kettle from the stove and put in a layer of clams cut in small pieces, then a layer of sliced potatoes alternately. Sprinkle on each layer salt, black pepper, sweet marjoram, a pinch of cayenne pepper and a little flour. Having saved all the water which was found in the clams, strain it and add with cold water enough to make a gallon. Return to the fire and let it boil slowly for an hour; then add five or six crackers, known as Pilot crackers, or even sea-biscuit, broken in pieces two or three inches in size, and let it cook a half hour longer.

